# *Francisella tularensis* Vaccines Elicit Concurrent Protective T- and B-Cell Immune Responses in BALB/cByJ Mice

**DOI:** 10.1371/journal.pone.0126570

**Published:** 2015-05-14

**Authors:** Roberto De Pascalis, Lara Mittereder, Alicia Y. Chou, Nikki J. Kennett, Karen L. Elkins

**Affiliations:** Laboratory of Mucosal Pathogens and Cellular Immunology, Division of Bacterial, Parasitic and Allergenic Products, Center for Biologics Evaluation and Research, U.S. Food and Drug Administration, Silver Spring, MD, United States of America; Midwestern University, UNITED STATES

## Abstract

In the last decade several new vaccines against *Francisella tularensis*, which causes tularemia, have been characterized in animal models. Whereas many of these vaccine candidates showed promise, it remains critical to bridge the preclinical studies to human subjects, ideally by taking advantage of correlates of protection. By combining *in vitro* intramacrophage LVS replication with gene expression data through multivariate analysis, we previously identified and quantified correlative T cell immune responses that discriminate vaccines of different efficacy. Further, using C57BL/6J mice, we demonstrated that the relative levels of gene expression vary according to vaccination route and between cell types from different organs. Here, we extended our studies to the analysis of T cell functions of BALB/cByJ mice to evaluate whether our approach to identify correlates of protection also applies to a Th2 dominant mouse strain. BALB/cByJ mice had higher survival rates than C57BL/6J mice when they were immunized with suboptimal vaccines and challenged. However, splenocytes derived from differentially vaccinated BALB/cByJ mice controlled LVS intramacrophage replication *in vitro* in a pattern that reflected the hierarchy of protection observed in C57BL/6J mice. In addition, gene expression of selected potential correlates revealed similar patterns in splenocytes of BALB/cByJ and C57BL/6J mice. The different survival patterns were related to B cell functions, not necessarily to specific antibody production, which played an important protective role in BALB/cByJ mice when vaccinated with suboptimal vaccines. Our studies therefore demonstrate the range of mechanisms that operate in the most common mouse strains used for characterization of vaccines against *F*. *tularensis*, and illustrate the complexity necessary to define a comprehensive set of correlates.

## Introduction

The investigational vaccine Live Vaccine Strain (LVS) is the only vaccine available in the United States to prevent tularemia, the disease caused by *Francisella tularensis* [1,2]. Although partial protection was obtained when LVS-vaccinated humans were challenged by aerosol with the most virulent Type A *F*. *tularensis* (subsp. *tularensis*) [3], the protection induced by LVS, which is derived from the less virulent type B *F*. *tularensis* (subsp. *holoartica*), against virulent *F*. *tularensis* is not fully understood. Vaccination of humans with other attenuated *Francisella* strains has resulted in some protection against tularemia in Russia, where Type B *Francisella* was endemic [4,5]. Although type A *Francisella* is present in the United States, tularemia does not represent a U.S. public health problem. However, *F*. *tularensis* is considered a category A bioterrorism agent because of the high infectivity and mortality rates following pulmonary infection [1]. Therefore, the development of a protective vaccine against type A *Francisella* is of interest.

Human clinical trials of vaccines against tularemia are impractical, due to the sporadic incidence of disease. However, new vaccines are being pursued through animal studies [6]. This approach depends on animal models that can efficiently bridge doses and efficacy to humans. Although mice are more susceptible to *F*. *tularensis* type B and *F*. *novicida* than humans, to date they have been the primary animal models for protection studies. Results from murine studies indicate that long term protection against *F*. *tularensis* infection, similarly to other intracellular pathogens, is largely due to T cell based immune responses [7–9]. However, high antibody titers have been quantified in both animals and humans following vaccination [10–13] or exposure [9,14]; in addition, B cell functions, in conjunction with T cell functions, are important for optimal rodent survival against LVS or *F*. *tularensis* (SchuS4) challenge [15–18], suggesting a protective role of B cells and/or humoral immune responses against *F*. *tularensis*.

Bridging protection from animal studies to human subjects is problematic due to the lack of reliable correlates of protection against *F*. *tularensis*. No mediators, including IFN-γ, correlate with protective T-cell immune responses [8]. Similarly, the measurement of serum antibodies has failed to correlate with protection against *F*. *tularensis* infection [9]. Using multivariate analyses, we recently proposed a model that reflects the complexity of the T cell immune response against intracellular bacteria, and which might overcome the lack of unique correlates of protection against *F*. *tularensis*. Initially, we adapted an *in vitro* tissue culture methodology [19] that measures the ability of splenocytes derived from vaccinated mice to control intramacrophage LVS bacterial growth. We further adapted this method to quantitate splenocyte gene expression [20]. Using a panel of vaccines of different efficacy, we demonstrated that both the ability to control intramacrophage bacterial replication by splenocytes and the expression of at least twelve selected genes correlated with the relative *in vivo* vaccine efficacy against lethal bacterial challenge. Some of the genes identified (e.g., IFN-γ and TNF-α) were previously demonstrated to be critical during LVS responses [7,8]. Subsequent studies in our lab validated the relevance of newly identified mediators, including T-bet [21], IL-12rβ2 [22], and IL-6 [23]. Similar functions and relative gene expression were also obtained with lymphocytes derived from *Francisella*-immune liver and lung tissues [24]. Moreover, when we integrated bacterial growth data with gene expression data, we distinguished vaccination groups quite well [24].

Nonetheless, while our studies suggest a promising approach, correlative cellular or humoral immune responses must be validated in different tissues and different animal species before bridging to humans. For example, whereas the overall hierarchy of expression was similar, we found that the relative levels of gene expression varied between cell types from different organs and by vaccination route [24]. Notably, all our findings to date have been obtained from analysis of lymphocytes from C57BL/6J mice, a Th1 dominant strain. Recently, it has been demonstrated that different mouse strains are not equally protected when immunized with LVS or with mutants of Type A *Francisella* [25]. In general, survival of vaccinated BALB/cByJ mice following challenge is greater than that of C57BL/6J mice, but most studies have been performed using only one mouse strain, limiting efficacy evaluations. These findings suggest that results using different mouse backgrounds for vaccination and protection studies may mislead the interpretation of vaccine efficacy.

In this report, we extended our studies by examining protection and the functions of splenocytes from another commonly used mouse strain, namely BALB/cByJ mice, to evaluate whether our correlate approaches apply to a different mouse strain. Because survival of BALB/cByJ mice was greater than that of C57BL/6J mice when they were vaccinated with LVS-derived vaccines, we assessed the role of humoral immune responses to determine whether antibodies play protective functions against lethal challenge in BALB/cByJ mice, especially following vaccination with suboptimal vaccines. We confirmed that our approach is a valid means to identify and quantify correlates of protection, and we demonstrated that T cell immune responses are comparable between mouse strains. In addition, we found B cell functions are important in BALB/cByJ mice vaccinated with suboptimal vaccines in protecting against LVS lethal challenge.

## Materials and Methods

### Experimental animals

Six to twelve week old wild type male BALB/cByJ, C57BL/6J, (Jackson Laboratories, Bar Harbor, ME), and B cell knockout (BKO) mice (Igh-J^tm1Dhu^, Taconic, Derwood, MD) were age-matched within each experiment. Here, BALB/cByJ mice were compared to BKO mice, as the exact substrain used for these mice was not available. All mice were housed in sterile micro isolator cages in a barrier environment, fed autoclaved food and water *ad libitum* and routinely tested for common murine pathogens by a diagnostic service of the Division of Veterinary Services, CBER. All experiments were carried out in accordance with the recommendations in the Guide for the Care and Use of Laboratory Animals of the National Institutes of Health, and were conducted under protocols approved by the Animal Care and Use Committee (ACUC) of CBER. Approved protocols provided scientifically validated humane endpoints, including pre-set criteria for euthanasia of moribund mice by CO_2_ inhalation. For each independent experiment, six-to-ten mice were vaccinated for each vaccine group. Six weeks after vaccination, three-to-five mice were sacrificed for spleen isolation. The remaining three-to-five mice were used for survival studies following lethal LVS challenge administration. The health status of the challenged mice was monitored and recorded twice a day. The stage when the mice did not markedly move, even in response to physical stimulus, and therefore were unable to reach water and food was considered a sign of imminent death. The mice were then sacrificed, without use of analgesic or anesthetics, and the time of sacrifice recorded as the time of death.

### Bacteria and growth conditions

*F*. *tularensis* LVS (American Type Culture Collection 29684), and *F*. *tularensis* LVS-G and LVS-R (both originally obtained from Dr. Francis Nano, University of Victoria, Victoria, British Columbia, CA), were grown to mid-log phase in modified Mueller-Hinton (MH) broth (Difco Laboratories, Detroit, MI) [26], harvested, and aliquots were frozen at -70°C. Numbers of live colony forming units (CFU), and intraperitoneal (IP) and intradermal (ID) LD_50_s were assessed as previously described [20]. In male BALB/cByJ mice, the ID LD_50_ is ~5x10^5^ CFU and the IP LD_50_ is < 5 CFU [27]. In addition, heat killed LVS (HK-LVS) was prepared immediately prior to use by treating aliquots of LVS at 60°C for 40 minutes; killing was confirmed by plating.

### Bacterial immunization and challenge

Parenteral ID immunizations were performed by administration of 1 x 10^4^ CFU LVS, 1 x 10^4^ CFU LVS-R, 1 x 10^4^ LVS-G or the amount equivalent to 1 x 10^8^ HK-LVS diluted in 0.1 ml phosphate-buffered saline (PBS) (BioWhittaker, Walkersville,MD); control groups received 0.1 ml PBS ID. Six weeks after vaccination, mice were challenged with 10^6^ LVS IP and monitored for survival. CFU of actual doses inoculated ID or IP were retrospectively confirmed by plate count; doses of each vaccine were optimized in initial experiments for maximal protection against lethal IP LVS challenge [20,24]. Mice were periodically bled by the tail vein for assessment of anti-LVS antibody production. Specifically, blood was collected before vaccination, two and six weeks after vaccination, and three days after lethal challenge; sera were pooled within each vaccination group.

### Preparation of lymphocytes and flow cytometry

Single-cell suspensions of splenocytes were generated for *in vitro* culture and flow cytometry by standard techniques, as previously described [20]. No detectable bacteria were found in organ homogenates at the time of harvest. Cell viability was assessed by exclusion of trypan blue, and by live/dead staining using a commercial available kit (Live/Dead Staining Kit; Invitrogen) and analysis by flow cytometry. Single cell suspensions prepared from spleens and splenocytes recovered from co-culture after the indicated time of culture were stained for a panel of murine cell surface markers and subjected to multiparameter analyses using a Becton-Dickinson LSR II flow cytometer (San Jose, CA) and FlowJo (Tree Star, Inc) software essentially as previously described [20].

### Co-culture of LVS infected bone marrow derived macrophages with splenocytes

Bone marrow derived macrophages (BMMΦ) were cultured in complete DMEM media supplemented with 10% L-929-conditioned medium. Confluent adherent macrophages were infected for 2 hours with LVS at a multiplicity of infection (MOI) of 1:20 (bacterium-to-BMMΦ), washed, treated for 1 hour with 50 μg/ml gentamicin, washed, and co-cultured with single-cell suspensions of splenic lymphocytes, derived from vaccinated and non-vaccinated control groups, in 24 well plates. Cells were harvested after 48 hr. and assessed for viability and for changes in cell surface phenotype by flow cytometry. Cells to be assessed for gene expression by qRT-PCR were pelleted, immersed in RNA*later* (Ambion, Austin, TX) and stored at -70°C until further characterization. Similarly, supernatants from harvested cells were stored at -70°C for further analyses. Adherent macrophages were lysed and intracellular bacteria loads determined, as previously described [20,24].

### Assessment of lymphocytes and supernatants

Non-adherent immune splenocytes from all groups were recovered on day two from each co-culture, and then analyzed in detail for relative gene expression by quantitative real time PCR. Total RNA extraction from samples (RNeasy mini kits, Qiagen, Valencia, CA), assessment by Bioanalyzer (Agilent Technologies, Santa Clara, CA), and cDNA synthesis (RetroScript Reverse Transcription for RT-PCR, Ambion, Applied Biosystems, Foster City, CA) were performed following the manufacturer’s instructions, as previously described [20]. Semi-quantitative real-time PCR was completed with a ViiA 7 sequence detection system (Applied Biosystems). cDNA synthesized from 20 ng of total RNA was diluted to a volume of 20 μl PCR mix (Applied Biosystems) containing sets of commercially available primers and probes (Applied Biosystem). Twenty-two genes, including twelve that were previously identified as potential correlates of protection against *F*. *tularensis* [20], and two housekeeping genes (GAPDH and Gusb) were analyzed. In addition, cDNA synthesized from 0.5–1 μg of total RNA was used to amplify a panel of genes involved in murine T-cell and B-cell activation, proliferation and differentiation (Rt2 Profiler PCR Array, Qiagen). Delta Ct (ΔCt) and the ratios between ΔCt of vaccine samples and control samples were then calculated.

Assessment of IFN-γ, TNF-α, IL-6, and IL-12 was performed in supernatants recovered on day two from *in vitro* co-cultures using standard sandwich ELISAs, according to the manufacturer’s instructions (BD Pharmingen, San Diego, CA); cytokines’ quantification was assessed by comparison to recombinant standard proteins (BD Pharmingen). Estimation of nitric oxide was performed in culture supernatants using the Griess reaction (Sigma-Aldrich, St Louis, MO; REF) and by comparison to serially diluted NaNO_2_.

### Assessment of humoral immune responses

Specific anti-LVS serum antibodies were determined by ELISA as described previously [28]. In brief, Immulon I plates were coated with 5 x 10^6^ bacteria per well of *F*. *tularensis* LVS diluted in 0.1M sodium bicarbonate solution and incubated for 2 hours at 37°C, then overnight at 4°C. The wells were then washed with PBS supplemented with 0.05% Tween 20 (PBST) and blocked with 10% bovine serum in PBS for 30 minutes at 37°C. Sera derived from vaccinated mice were serially diluted in PBST with 10% fetal bovine serum, added to each well and incubated for 90 minutes at 37°C. The plates were washed with PBST and incubated with horseradish peroxidase conjugated antibodies detecting mouse total IgG, or IgG isotypes, or IgM (Southern Biotech, Birmingham, AL) diluted 1:3000 in PBST with 10% bovine serum for 90 minutes at 37°C. The assay was developed by the addition of ABTS peroxidase substrate (Kirkegaard & Perry Laboratories, Gaithersburg, MD) and 30 minutes’ incubation at room temperature protected from light. Optical density was read at 410 nm, reference 630 nm, with a microplate reader (Molecular Devices, Sunnyvale, CA). Endpoint titers were determined by selecting the dilution factor at which the average sample O.D. minus one standard deviation was greater than the average O.D. of the naïve sample, plus 3 standard deviations, as well as an O.D. value > 0.100.

### Assessment of antigen expression

Protein content of vaccine preparations was quantitated by BCA Protein Assay kit (Pierce, Rockford, IL). Twenty μg of protein extract from whole LVS, LVS-G, LVS-R and HK-LVS bacteria, lysed in 1% SDS, were electrophoresed on a 4–12% Bis-Tris SDS-PAGE gel (Life Technologies) and transferred to a nitrocellulose membrane. Immediately after protein transfer, the membranes were washed with Ponceau S concentrate (Sigma) for 3 minutes. The membranes were then washed with distilled water for 3 minutes, and images of the stained membranes were acquired. Membranes were then blocked with 5% Blocking Grade Non-Fat Dry Milk (Bio-Rad) in Tris-Buffered Saline with 0.1% Tween 20 (TBST). Pooled sera, collected after two or six weeks from mice vaccinated with LVS or heat-killed LVS, or collected after three days after LVS challenge, were used as primary antibodies at a 1:1000 dilution in TBST. A conjugated anti-mouse IgG antibody (Sigma-Aldrich) at a 1:4000 dilution in TBST was used to detected antigen binding antibodies. Blots were developed with BCIP/NBT Phosphatase substrate (Kirkegaard & Perry Laboratories) for 15 minutes, after which the reaction was stopped with water, per the manufacturer’s instructions.

## Results

### *In vivo* protection and *in vitro* co-culture determination of lymphocyte activities against *Francisella tularensis* LVS

To determine whether the *in vitro* co-culture system reflects the *in vivo* protection in BALB/cByJ mice, we first evaluated protection in this strain using the panel of vaccines that provided different degrees of protection against lethal *Francisella* challenge in C57BL/6J mice [20]. BALB/cByJ mice were vaccinated ID with LVS, with the opacity variants LVS-G and LVS-R, or with heat killed (HK-) LVS, and then challenged four to six weeks after vaccination with the largest available lethal dose of LVS IP (10^6^ CFU) (Table 1). All mice vaccinated with wild type LVS survived this challenge dose. Excellent protection was observed also in mice vaccinated with the two other live attenuated vaccines, LVS-R and LVS-G, and, surprisingly, in mice vaccinated with HK-LVS. In particular, from an average of seven individual survival experiments, animals vaccinated with LVS-G exhibited 97% survival following challenge; mice vaccinated with LVS-R exhibited 70% survival; and vaccination with HK-LVS provided 82% protection. These data indicated that most vaccinated BALB/cByJ mice survived lethal LVS challenge, and thus the panel of vaccines did not exhibit the hierarchy of relative protection that was observed in C57BL/6J mice (Table 1) [20].

**Table 1:** Effect of differential vaccination on survival against lethal challenge with LVS.

	BALB/cByJ	BKO	C57BL/6J
Vaccine	SurvivalChallenge IP	Survival	Survival
**Naïve**	**0%**	**0%**	**0%**
**LVS**	**100%**	**100%**	**100%**
**LVS-G**	**97% (80–100)**	**60% (20–80)**	**71% (33–100)**
**LVS-R**	**70% (20–100)**	**40% (0–80)**	**47% (0–80)**
**HK-LVS**	**82% (40–100)**	**7% (0–25)**	**35% (0–66)**

Three to five BALB/cByJ, BKO, or C57BL/6J mice per group, for a total of 33–35, 14–15, or 15–17 mice per vaccine group, respectively, were vaccinated I.D. with the indicated vaccines, and then challenged 4–6 weeks later with a lethal dose of 10^6^ LVS I.P. Values represent combined results of seven, three, or four-five independent experiments for the BALB/cByJ, BKO, or C57BL/6J mice, respectively. Values in parenthesis indicate ranges of survival among experiments.

**Fig 1:**
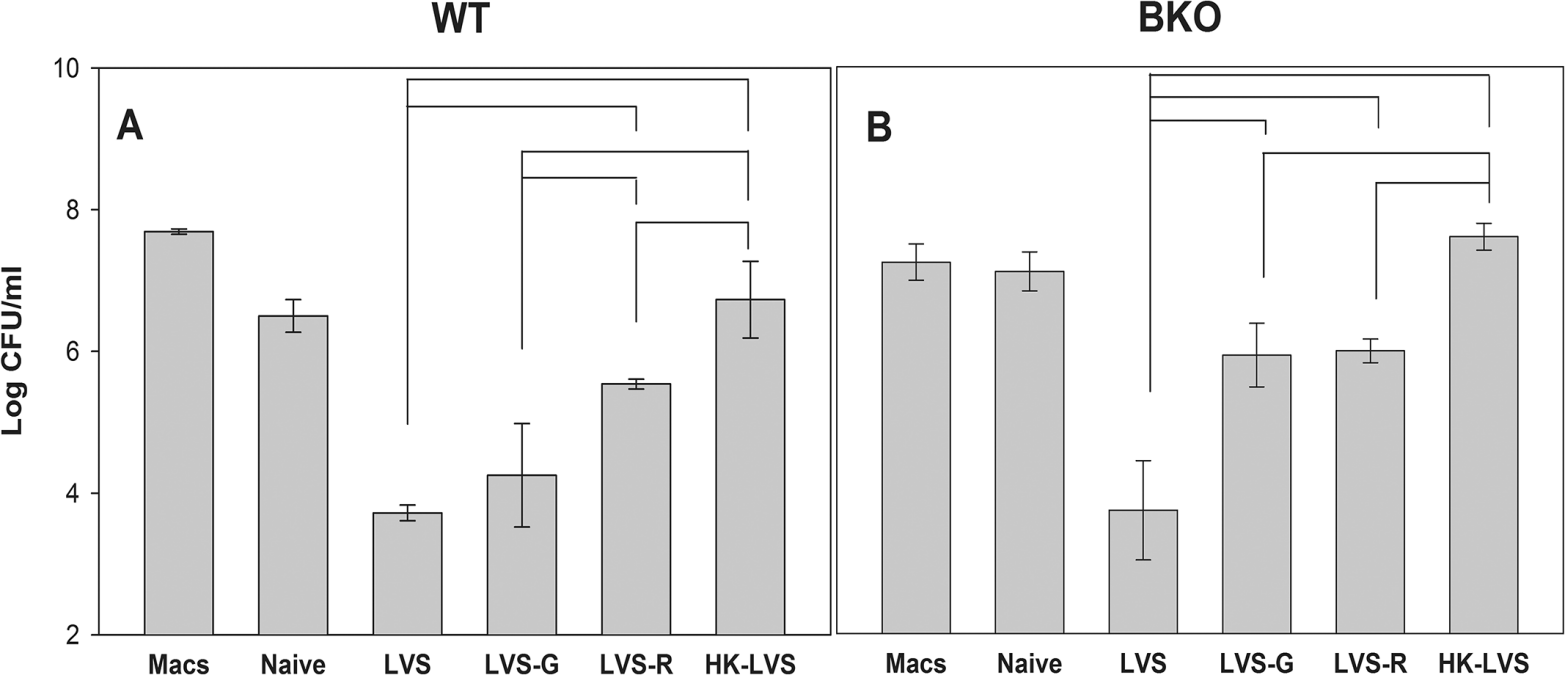
Splenocytes from LVS-related vaccinated mice exhibit a hierarchy of control of intramacrophage LVS growth. BMMΦ from BALB/cByJ mice were infected with LVS (Macs), and co-cultured with splenocytes obtained from naïve or vaccinated BALB/cByJ mice (Panel A), and from naive or vaccinated BKO mice (Panel B), as indicated. After two days of co-culture, BMMΦ were washed, lysed, and plated to evaluate the recovery of intracellular bacteria. Values shown are the mean numbers of CFU/ml ± SD of viable bacteria for triplicate samples. Results shown are from one representative experiment of seven (using splenocytes of BALB/cByJ mice) or four (using splenocytes of BKO mice) independent experiments of similar design and outcome. Brackets indicate a significant difference (*P* < 0.05) between the recoveries of bacteria in co-cultures. There were no significant differences between the recovery of bacteria from co-cultures using LVS-immune cells and LVS-G-immune cells (Panel A) and the recovery of bacteria from co-cultures using LVS-G-immune cells and LVS-R-immune cells (Panel B).

In parallel with *in vivo* vaccination and challenge studies, single cell suspensions from spleens were prepared from mice vaccinated with LVS, LVS-G, LVS-R, HK-LVS, or from naive mice, and their activities were compared using *in vitro* co-culture studies. As shown in S1 Table, panel A, analyses of the distribution of input splenocytes did not reveal any obvious differences between naïve and vaccinated mice. After two days of co-culture, approximately 20–40% of the input number of splenocytes was recovered in all groups; of those, about 60% were live cells (S1 Table, panel B). In addition, flow cytometry analyses revealed that the majority of splenocytes recovered were B and T cells, whereas the non-B or non-T cells were strongly reduced compared to the input cells. As consequence, CD4^+^ T cells were relatively enriched, particularly in the LVS and LVS-G vaccinated groups (S1 Table, panel B). Functionally, Fig 1, panel A shows that cells obtained from LVS-infected mice were most effective in controlling the intramacrophage growth of LVS, while those from LVS-G and LVS-R vaccinated mice were less effective. Cells derived from HK-LVS vaccinated mice were the least effective, resulting in no bacterial growth inhibition, with numbers of recovered bacteria comparable to those from cultures with naive cells or cultures with LVS-infected macrophages only. In contrast to the *in vivo* survival data, the hierarchy of *in vitro* activities of cells from vaccinated BALB/cByJ mice was similar to that obtained with cells from vaccinated C57BL/6J mice: LVS > LVS-G > LVS-R > HK-LVS [20].

**Fig 2:**
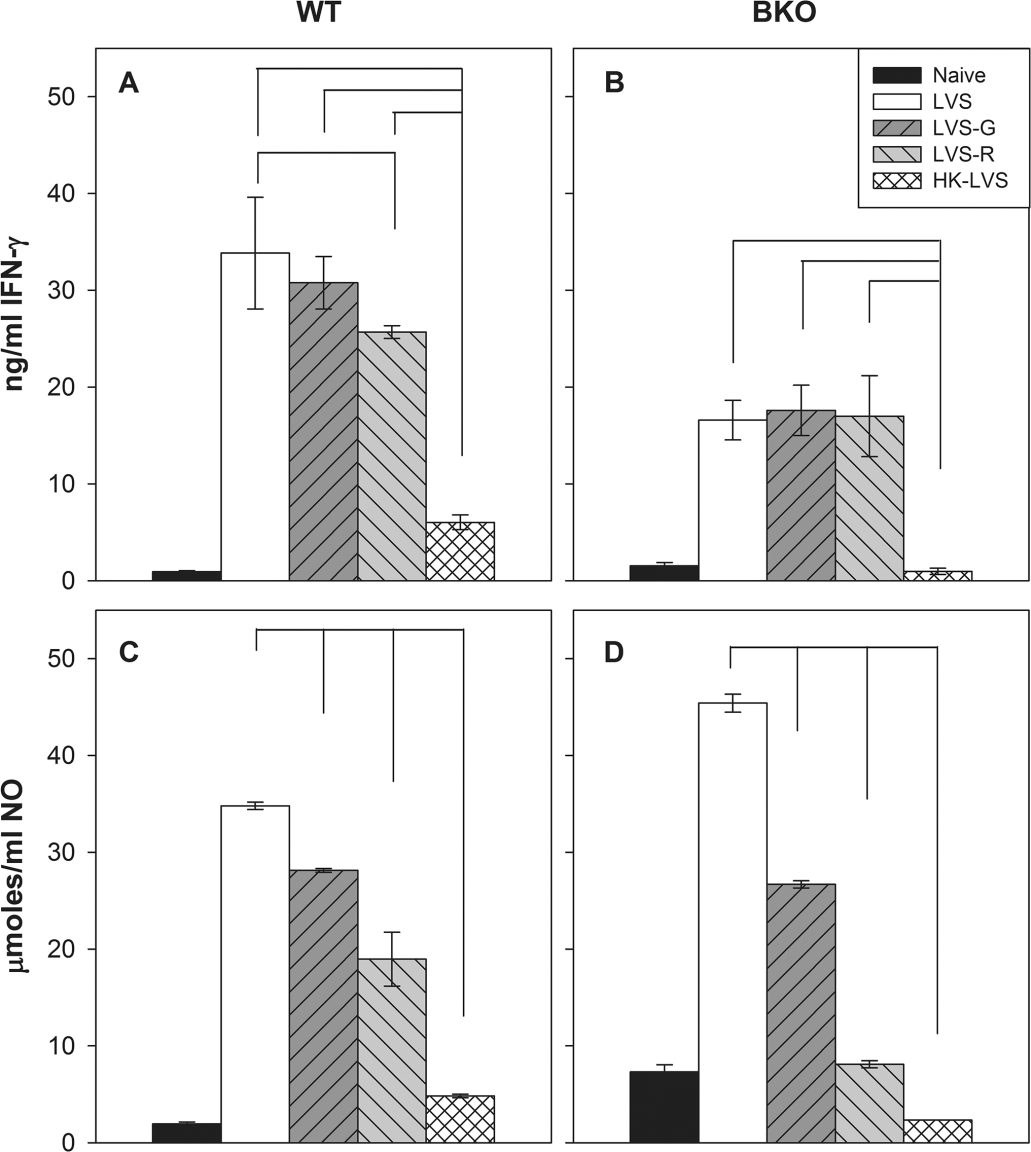
IFN-γ and NO production exhibit patterns mostly similar to that of *in vitro* LVS replication. Supernatants from co-cultures described in Fig 1 using splenocytes of BALB/cByJ mice (Panels A and C) and BKO mice (Panels B and D) were collected after two days of co-culture, and separated from cells for analyses of IFN-γ by ELISA (Panels A and B) and NO by Griess reaction (Panels C and D). Concentrations were calculated using standard curves as reference. Values shown are the mean concentration in ng/ml (IFN-γ) or μmoles (NO) ± standard deviation of triplicate samples. Results shown are from one representative experiment of seven (using splenocytes of BALB/cByJ mice) or four (using splenocytes of BKO mice) independent experiments of similar design and outcome. Brackets indicate a significant difference (*P* < 0.05) between amounts of IFN-γ or NO produced in co-cultures. There were no significant differences in IFN-γ production between the co-cultures using LVS-immune cells and the co-cultures using LVS-G-immune cells (Panel A), between the co-cultures using LVS-G-immune cells and the co-cultures using LVS-R-immune cells (Panel A), and between the co-cultures using LVS-immune cells and the co-cultures using LVS-G-immune cells or LVS-R-immune cells (Panel B).

Supernatants and cells were recovered after 2 days of *in vitro* co-culture and further analyzed. Analysis of supernatants demonstrated that the relative amounts of IFN-γ and NO production (Fig 2, panel A and C), as well as TNF-α and IL-12 p40 production (S1 Fig, panels A and B), exhibited similar patterns as those observed in *in vitro* control of intramacrophage LVS replication: co-cultures with splenocytes from LVS-vaccinated mice produced the highest amounts of each, followed by LVS-G, LVS-R, HK-LVS and naive groups. Increased IL-6 production was associated with all vaccine groups (S1 Fig, panel C). Collectively, these data indicated that the absence of hierarchy of protective capacity engendered by *in vivo* vaccination with this panel of vaccines in BALB/cByJ mice was in contrast to the hierarchy of each type of *Francisella*-immune splenocytes in producing relevant cytokines and nitric oxide, and ultimately in differential control of intramacrophage bacterial growth.

### Relative gene expression during co-culture by *Francisella-immune* splenocytes after differential vaccination of BALB/cByJ mice

Because cytokines, nitric oxide production, and control of intramacrophage bacterial growth of these *in vitro* co-culture conditions detected differences in vaccine quality, we focused our analysis on the twelve genes whose relative expression best reflected the hierarchy of *in vivo* vaccine efficacy in C57BL/6J mice [20]. We further analyzed CCR5 gene expression; CCR5 was the only gene, among an additional ten genes that were individually analyzed [20], that in BALB/cByJ mice showed a differential gene expression pattern among vaccine groups in exploratory experiments. Non-adherent immune splenocytes from all groups were recovered and analyzed for relative gene expression by quantitative real time PCR (Table 2). As observed using immune splenocytes from C57BL/6J mice, splenocytes from BALB/cByJ mice exhibited relative gene expression patterns that were correlated with *in vitro* activities. In particular, the expression of IL-18bp, IL-27, CCR5, and to a lesser degree IL-12rβ2 was higher in cells derived from LVS and LVS-G-vaccinated mice, followed by lesser expression in splenocytes from LVS-R and HK-LVS-vaccinated mice, as compared to non-vaccinated naive mice. In addition, a few genes, including IFN-γ, GM-CSF, T-bet, and SOCS-1, were more strongly up-regulated in splenocytes from LVS, LVS-G and LVS-R vaccinated mice than in cells from HK-vaccinated mice. The degree of differences between vaccine groups was smaller for the remaining genes. Overall, these data confirmed the discrepancy between the *in vivo* survival data from BALB/cByJ mice and the *in vitro* data, similar to that observed from the cytokine and nitric oxide production, and the control of intramacrophage bacterial growth.

**Table 2:** Relative gene expression of potential correlates in co-cultures using splenocytes from differentially vaccinated BALB/cByJ, BKO, and C57BL/6J mice.

	BALB/cByJ				BKO				C57BL/6J			
	LVS	LVS-G	LVS-R	HK-LVS	LVS	LVS-G	LVS-R	HK-LVS	LVS	LVS-G	LVS-R	HK-LVS
**IFN-γ**	43	59	56	2.0	16	43	76	0.6	89	93	89	0.9
**TNF-α**	1.7	1.8	1.8	1.2	2.3	1.9	1.9	1.2	1.9	1.8	1.6	0.8
**IL-6**	1.2	1.5	1.9	1.9	0.1	0.7	0.7	0.7	2.7	6.1	4.3	0.9
**IL-12rβ2**	2.8	2.1	2.1	1.2	1.0	2.4	2.2	0.9	5.0	3.6	4.5	1.2
**IL-18bp**	6.8	4.0	1.9	1.0	5.9	3.3	2.9	0.9	3.2	2.1	2.6	1.1
**IL-27**	9.2	4.4	3.7	1.3	3.5	2.9	2.7	1.0	2.5	3.0	2.7	0.9
**GM-CSF**	3.3	3.9	5.2	1.3	1.4	4.6	7.5	0.6	9.1	5.6	6.0	0.9
**T-bet**	2.9	3.2	3.8	1.4	2.9	2.1	2.4	0.8	4.4	4.0	3.5	1.1
**IL-22**	1.0	1.4	2.3	1.6	0.5	1.0	2.2	0.7	2.7	1.7	4.1	0.9
**CCL7**	0.6	1.0	1.2	1.1	0.3	0.7	0.9	0.6	0.8	0.9	0.4	1.0
**Irf-1**	1.2	1.2	1.5	1.2	2.3	2.1	1.3	1.1	1.3	1.4	1.4	1.0
**SOCS-1**	2.1	2.2	3.0	1.6	2.4	1.3	2.5	0.9	2.7	1.6	2.1	1.2
**CCR5**	6.55	6.9	3.3	1.2	9.55	4.7	2.9	1.5	2.4	1.3	1.3	1.2

Values indicate median fold change of the indicated genes, compared to naive cells; values derived from analyses of splenocytes of BALB/cByJ, BKO, and C57BL/6J mice were calculated from seven, four, and four independent experiments, respectively.

Further, because these *in vitro* activities are related to T cell functions [19], these data suggest that the T cell immune responses of BALB/cByJ mice are comparable to those of C57BL/6J mice. However, some differences were observed between the relative gene expression of splenocytes from differentially vaccinated BALB/cByJ and C57BL/6J mice. In particular, relative levels of TNF-α, IL-6, and IL-22 were not well differentiated in BALB/cByJ mice compared to C57BL/6J mice. With the exception of CCL7 and Irf1, the data obtained using splenocytes of C57BL/6J mice in experiments concurrent with those in BALB/cByJ mice were similar to those obtained previously [20], and suggested weak T cell immune responses in HK-LVS-vaccinated BALB/cByJ mice. However, since most HK-LVS BALB/cByJ vaccinated mice survived IP challenge with LVS, other factors, such as B cell functions, may compensate for weak T cell immune responses and contribute to the protection of BALB/cByJ mice vaccinated with suboptimal vaccines.

### Role of B cells and humoral immune responses in protection following differential vaccination of BALB/cByJ mice

To evaluate B cell functions, we initially analyzed T-cell and B-cell activation profiles by a PCR array system. Several genes involved in T- or B-cell activation, proliferation and/or differentiation had similar expression patterns in splenocytes from BALB/cByJ and C57BL/6J vaccinated mice (S2 Table). However, genes involved in B cell activation, proliferation, or differentiation were mostly up-regulated in LVS and LVS-R vaccinated mice, but not in HK-LVS vaccinated BALB/cByJ mice. This suggests that either splenocytes from HK-LVS vaccinated mice are not reactive to *in vitro* LVS stimulation, or that B cell functions are weaker in HK-LVS than in LVS and LVS-R vaccinated BALB/cByJ mice.

To directly evaluate the role of B cell functions, BKO mice on a BALB/cByJ background were vaccinated with the panel of vaccines. Six weeks after vaccination, mice were either sacrificed for *in vitro* studies or challenged with a large lethal dose of LVS for survival studies. For *in vitro* studies, splenocytes from the different vaccine groups were co-cultured with LVS-infected macrophages, and were then recovered for mRNA analyses. The data revealed that BKO mouse-derived splenocytes controlled bacterial replication in patterns similar to that of BALB/cByJ wild type splenocytes (Fig 1, panel B). Immune splenocytes from the LVS group controlled bacterial replication more efficiently than those from the LVS-R and HK-LVS groups. Splenocytes from LVS-G-vaccinated BKO mice controlled bacterial replication similarly to that of the LVS-R group. The analyses of IFN-γ production from co-cultures revealed that LVS, LVS-R and LVS-G vaccination groups produced significantly higher amounts of IFN-γ compared to that of HK-LVS group (Fig 2, panel B). High production of nitric oxide in the LVS group, followed by the LVS-G, LVS-R, and HK-LVS groups, was observed in supernatants of co-cultures using splenocytes from vaccinated BKO mice (Fig 2, panel D). The relative gene expression of the potential correlates of protection from splenocytes recovered from co-cultures again demonstrated the similarities between WT and BKO mice (Table 2). IL-18bp, IL-27, and CCR5 genes were expressed at higher levels in the LVS group, followed by LVS-G, LVS-R, and HK-LVS; in contrast, IL-12rβ2 and GM-CSF were not upregulated in the LVS group, but they were in the LVS-G and LVS-R groups. Similar to the WT mice, IFN-γ, T-bet, and SOCS-1 were upregulated in all vaccine groups, but not in the HK-LVS group.

In parallel with the *in vitro* studies, vaccinated BKO and WT mice were challenged with a lethal dose of LVS to evaluate the vaccines’ protection. Table 1 shows that, while LVS vaccinated BKO mice were fully protected, the other groups suffered a reduction of protection compared to WT mice. In particular, on average only 7% of HK-LVS vaccinated BKO mice survived, compared 82% of the WT mice.

Overall, the *in vitro* data indicate that T cell immune responses are mostly similar between BALB/cByJ WT and BKO mice. However, the *in vivo* studies suggest that the lack of B cells compromised the survival of BKO mice vaccinated with the sub-optimal vaccines. Interestingly, the overall survival rate of BKO mice resembles closely that of C57BL/6J (Table 1) [20], suggesting that the improved protection in vaccinated BALB/cByJ mice compared to C57BL/6J may be due to B cell functions.

**Fig 3:**
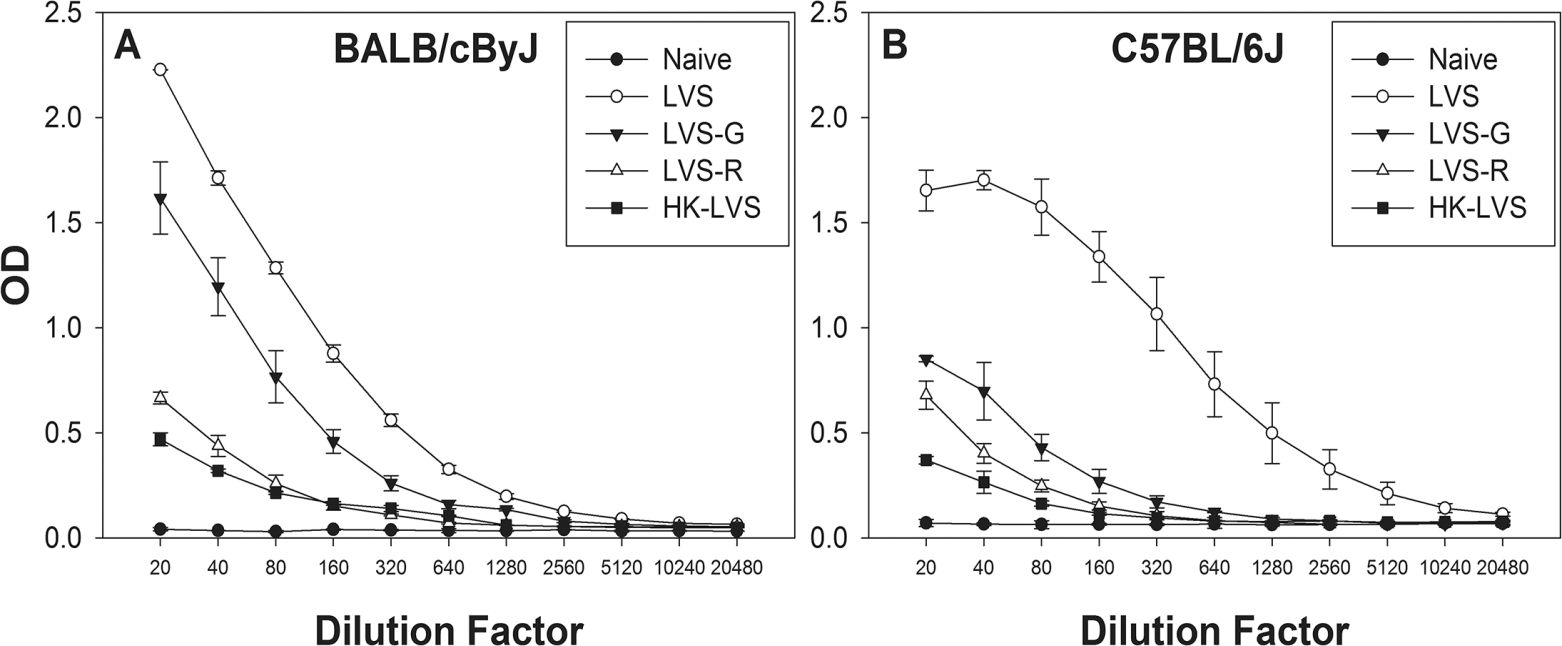
Humoral immune responses patterns to LVS-related vaccines differ between BALB/cByJ and C57BL/6J mice. Pooled sera from five mice for each vaccine group were obtained from BALB/cByJ mice (Panel A) and from C57BL/6J mice (Panel B) six weeks after vaccination, and analyzed for anti-LVS total IgG. Error bars depict standard deviation of the mean of triplicates samples tested in the ELISA. Results shown are from one representative experiment of four independent experiments of similar design and outcome.

To evaluate whether the improved protection of BALB/cByJ mice compared to C57BL/6J mice was due to humoral immune responses, sera from LVS-derived vaccinated mice were obtained before vaccination, two and six weeks after vaccination, and three days after lethal challenge. Sera from each vaccine group were pooled and analyzed for total anti-LVS IgG, IgG_1_, IgG_2a_, IgG_2b_, IgG_2c_, IgG_3_, and IgM. Overall, vaccination of BALB/cByJ or C57BL/6J mice with LVS induced the highest levels of specific antibody production, followed by that of LVS-G vaccinated mice. LVS-R and HK-LVS-vaccinated and C57BL/6J mice had the lowest antibody titers. Fig 3 shows comparative profiles between mouse strains of total IgG analyses by ELISA at 6 weeks after vaccination. At this time point, as well as three days after lethal challenge, the differences between mouse strains were noticeable, especially in the HK-LVS vaccine group (S3 Table). This vaccine induced higher specific antibody production in BALB/cByJ mice than C57BL/6J mice. In contrast, mice vaccinated with LVS, LVS-G, or LVS-R did not exhibit any differences between mouse strains at any time point. In addition, Fig 3 illustrated differences in the IgG curve profiles between mouse strains. In particular, only the total IgG of C57BL/6J mice vaccinated with LVS seemed to reach a plateau at the lowest dilutions. These results suggest the presence of different anti-LVS antibody sub-populations and/or different affinities of binding of the antibodies generated by the different mouse strains. More detailed analyses of amounts of antibody isotypes, including IgG_1_, IgG_2a_, IgG_2b_, IgG_2c_, IgG_3_, and IgM, revealed some differences between BALB/cByJ mice and C57BL/6J mice (S4 Table). In particular, IgG_1_ and IgG_2b_ titers were generally higher in BALB/cByJ mice and C57BL/6J mice, respectively. However, we observed substantial variability between replicate experiments, and no clear pattern of informative differences between vaccine groups.

**Fig 4:**
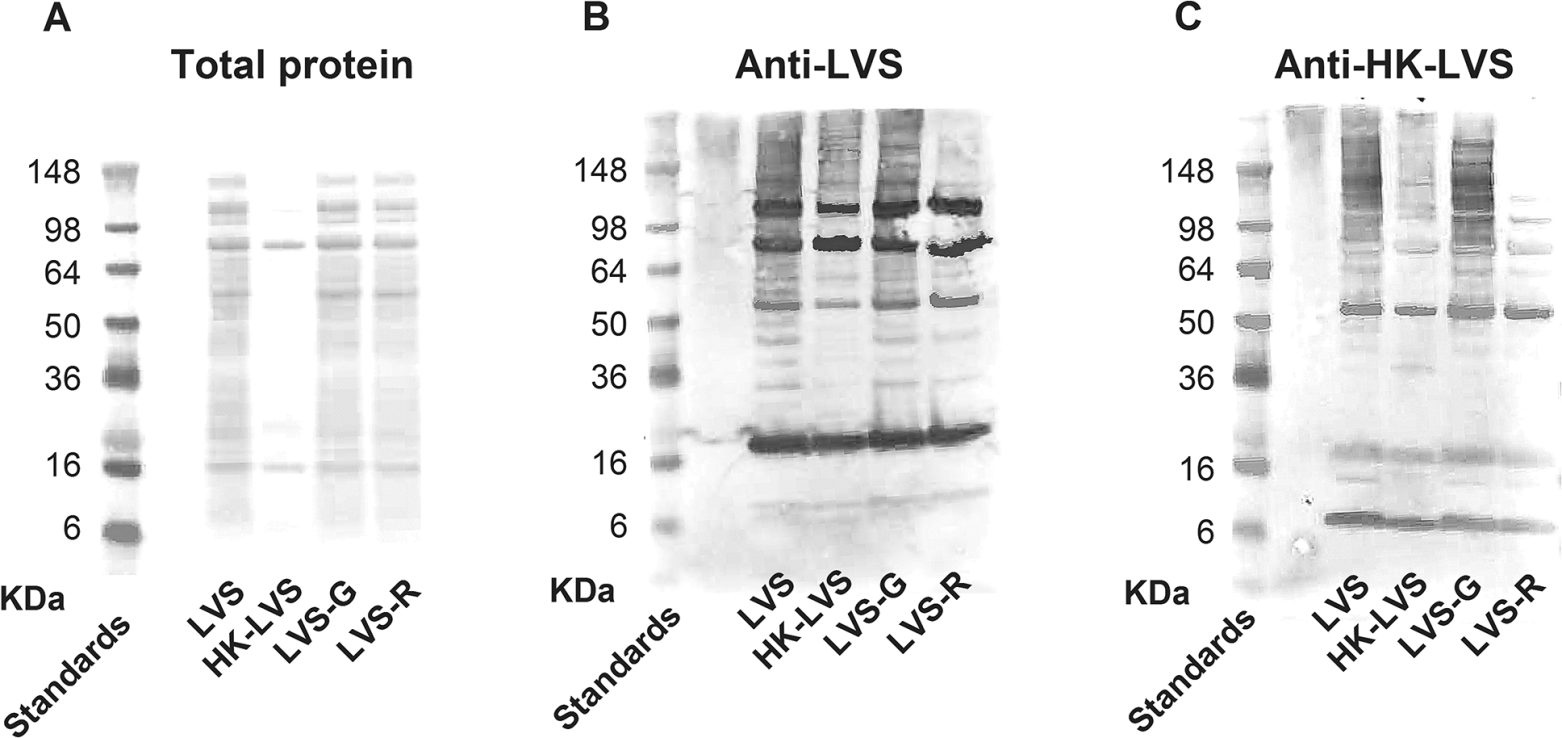
Protein staining and reactivity with serum derived from vaccinated mice revealed differences among vaccine extracts. Twenty μg of protein extracts, prepared from whole LVS, HK-LVS, LVS-G, and LVS-R, were loaded on SDS-PAGE in reducing conditions and stained with Red Ponceau (Panel A). Following Ponceau staining, reactivity of protein extracts was analyzed by blotting the membranes with pooled sera derived from LVS vaccinated (Panel B) and from HK-LVS vaccinated BALB/cByJ mice (Panel C). Results shown are from one representative experiment of four independent experiments of similar design and outcome.

To evaluate whether LVS-derived vaccines may express different antigens compared to LVS and therefore elicit different populations of specific antibodies, sera derived from vaccinated BALB/cByJ mice were analyzed by Western blot. Initially, whole bacterial extracts prepared from LVS, LVS-G, LVS-R and HK-LVS strains were loaded on SDS-PAGE in reducing conditions and analyzed for protein content after Ponceau staining (Fig 4, panel A). The comparative analyses of the major bands did not reveal any obvious differences between the bacterial strains LVS, LVS-G, and LVS-R. In contrast, HK-LVS showed an overall reduction in the number of bands, likely due to protein degradation and/or aggregation, accompanied by reduced detection on this type of gel. The HK-LVS sample contained no additional protein bands compared to the other samples. Subsequently, sera derived from LVS and HK-LVS vaccinated mice were used in parallel as primary antibodies to evaluate antigen expression by the three live vaccines and the HK-LVS vaccine. When sera derived from LVS-vaccinated mice was used to blot the membrane (Fig 4, panel B), no major differences were detected between the bacterial strains LVS and LVS-G. In contrast, HK-LVS and LVS-R showed fewer bands and a weaker intensity of the bands. Similarly, when sera derived from HK-LVS-vaccinated mice was used as the primary antibody in the Western blot (Fig 4, panel C), the LVS and LVS-G antigenic profiles appeared comparable, whereas HK-LVS and LVS-R exhibited fewer and weaker bands. Obvious differences between the two Western blots were represented by the increased intensity of the bands sizing approximately 16 kDa and 6 kDa when probed with anti-HK-LVS sera. However, these findings were consistent among vaccine groups. Overall, these data correlate with the quantification of total IgG (S3 Table), confirming that LVS and LVS-G induce higher humoral immune responses than LVS-R and HK-LVS in BALB/cByJ mice.

**Fig 5:**
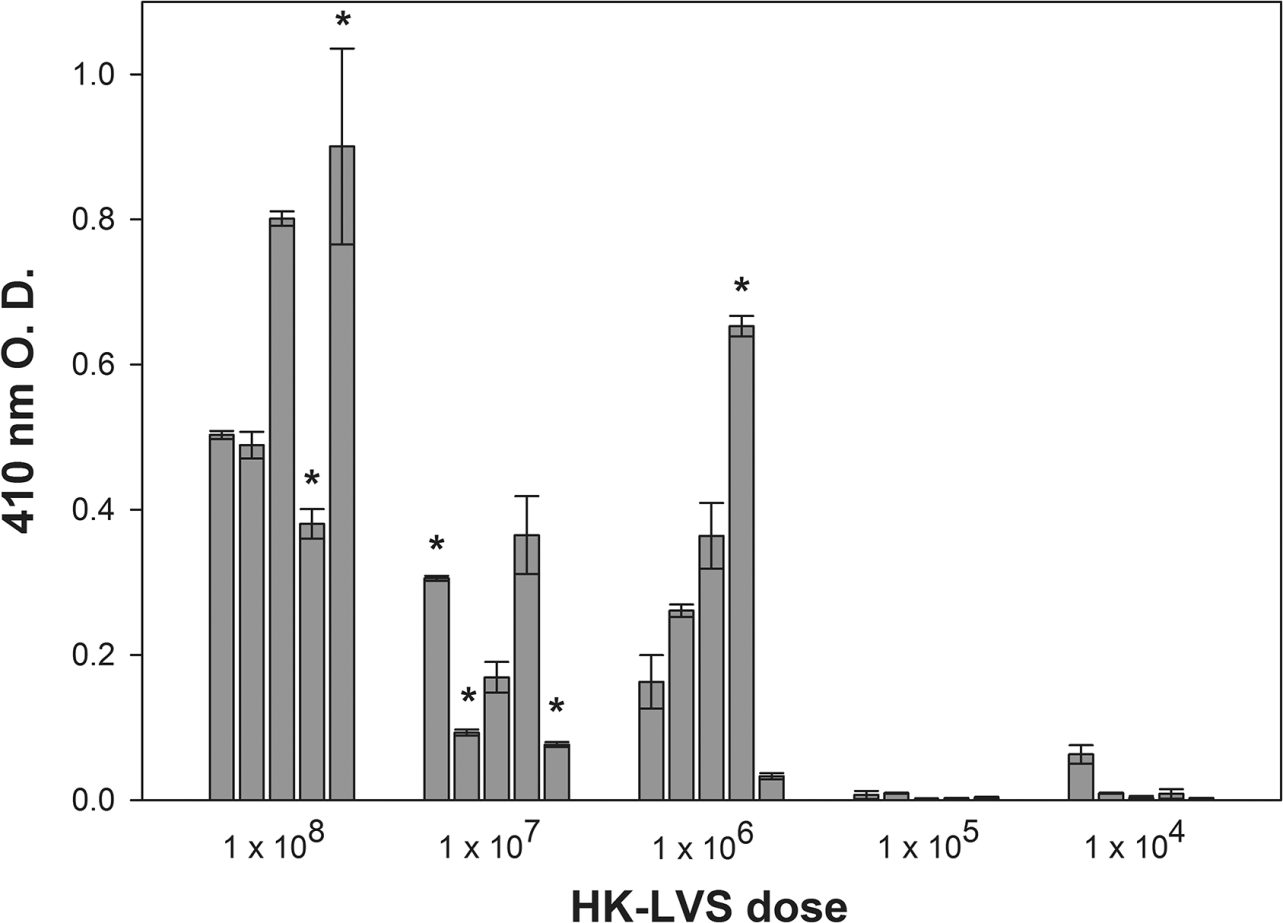
Anti-LVS antibody titers of HK-LVS vaccinated BALB/cByJ mice do not correlate with survival. Sera from BALB/cByJ mice vaccinated with different doses of HK-LVS were individually analyzed six weeks after vaccination for anti-LVS total IgG. Mice were then challenged IP with a maximal lethal dose of LVS. Error bars depict standard deviation of the mean of triplicates samples tested in the ELISA. * indicates the antibody responses of the mice that eventually survived the lethal challenge. Results shown are from one representative experiment of two independent experiments of similar design and outcome.

The overall survival of HK-LVS vaccinated BALB/cByJ mice, when vaccinated with 10^8^ HK-LVS and then challenged with a lethal dose of LVS, was 82%. To evaluate whether effectiveness of the vaccination was related to the dose administered, BALB/cByJ mice were vaccinated with different doses of HK-LVS. Results from two independent experiments showed that the overall survival and time of death correlated with the vaccination doses. All mice vaccinated with 10^4^–10^5^ HK-LVS died within 4–6 days after lethal challenge. In contrast, 20% and 40% of mice vaccinated with 10^6^ and 10^7^ HK-LVS, respectively, survived the LVS challenge, and the time of death of those that died was extended. These data indicate that the induction of B cell immune responses requires a high antigenic stimulation, which is provided by replicating live vaccines or a high dose of killed LVS. Interestingly, whereas antibody titers were overall higher in mice that received higher vaccination doses, there was no direct correlation between survival and total IgG antibody titers (Fig 5).

## Discussion

The 2001 terrorist and anthrax bioterrorist attack in the United States prompted research and interest in the generation and characterization of novel vaccines against the Category A agent *F*. *tularensis*. The genes responsible for attenuation of LVS have been approximately identified, and the size of the regions affected leads the authors to conclude that it is unlikely LVS could revert to virulence [29]. In addition, newly manufactured LVS has been reported to be well tolerated in rabbits and humans [30,31]. However, LVS has not been licensed in the U.S. and the extent of its protection against challenge of humans with type A *F*. *tularensis* appear to be partial [2,3,32–34]. Several LVS-derived mutants have been generated, and many of them have demonstrated to have a protective capacity in mice against lethal challenge with LVS comparable to that of LVS vaccinated mice [35]. In addition, several mutants derived from fully virulent Type A *F*. *tularensis* (SchuS4) are highly protective against low doses of IN SchuS4 challenge in C57BL/6J mice [36], and in BALB/cByJ mice [25,36,37] or against ID or SC SchuS4 challenge in BALB/cByJ mice [25,38,39]. Interestingly and paradoxically, better protection against challenge with fully virulent *Francisella* after vaccination has generally been found when using BALB/cByJ mice, a Th2 dominant mouse strain, particularly after aerosol challenge. In a direct comparison, one of the most promising vaccines, namely Δ*clpB* mutant, protected BALB/cByJ mice but not C57BL/6J mice against aerosol SchuS4 challenge [25]. These results highlight the need for animal models that best resemble human immune responses against *F*. *tularensis*.

Previously, we have described a method that discriminated vaccines of different efficacies. This approach combined data derived from *in vitro* control of intramacrophage bacterial replication by lymphocytes from spleens of vaccinated mice with gene expression data from the same lymphocytes. These functions are largely due to immune T cell activities [19]. In addition, we demonstrated that these functions were also present in lymphocytes derived from liver and lung as well as spleen; relative gene expression varied slightly according to the vaccination route and cell types from different organs. Our findings were first obtained using C57BL/6J mice, a Th1 dominant mouse strain. Although Th1 T cell responses are associated with control of intracellular pathogens, it has proved to be more difficult to induce protective immunity against *F*. *tularensis* in C57BL/6J mice than in BALB/cByJ mice, an issue that remains under study elsewhere. These results pointed out the need to extend our studies to other mouse strains to further assess our analytical approach.

Initially, we evaluated the *in vivo* efficacy in BALB/cByJ mice of the four vaccines, namely LVS, LVS-G, LVS-R, and HK-LVS. Six weeks following vaccination, BALB/cByJ mice were challenged with a lethal IP injection of LVS. Overall, survival rates were higher in all vaccination groups in BALB/cByJ mice compared to C57BL/6J mice. Similar to the data obtained using C57BL/6J mice, LVS and LVS-G were the most efficient vaccines in BALB/cByJ mice. LVS-R vaccinated mice were protected at a rate slightly higher than the C57BL/6J counterparts (70% vs ~ 50%). Vaccination with HK-LVS showed the highest difference between C57BL/6J and BALB/cByJ mice: about 80% of BALB/cByJ mice survived the lethal challenge, compared to a smaller proportion of the C57BL/6J mice. These results suggested that additional immune responses in BALB/cByJ mice minimized the different protective capacities between vaccines observed in C57BL/6J mice.

In contrast with the *in vivo* data, the *in vitro* results demonstrated that the splenocytes’ activities reflected the vaccine efficacy and *in vitro* activities observed in C57BL/6J mice [20,24]. The hierarchy of vaccines’ efficacy, LVS > LVS-G > LVS-R > HK-LVS, was mirrored here in the ability of splenocytes of vaccinated BALB/cByJ mice to control *in vitro* LVS replication (Fig 1), as well as in the production of selected cytokines and nitric oxide from the co-culture supernatants (Fig 2, S1 Fig, Table 2, and S2 Table). Although splenocytes mostly contain B and T lymphocytes, after 48 h of co-culture we observed a relative enrichment of T cells, particularly in the LVS and LVS-G groups (S1 Table). These findings, together with the knowledge that B cell depletion does not alter *in vitro* activities [19], suggest that T cell immune responses in BALB/cByJ mice are comparable to that of C57BL/6J mice. Similar to the findings using C57BL/6J mice, differences between groups were more evident between the live vaccines (LVS, LVS-G, LVS-R) and HK-LVS, but more subtle among the live vaccines, particularly in the analysis of protein and gene expressions (e.g., IFN-γ). This further supports our proposed approach of quantifying correlates of protection by combining data derived from multiple assays [24]. We next focused on analyzing the expression of those genes that were originally selected by screening splenocytes of C57BL/6J mice vaccinated with LVS-derived vaccines [20], many of which have previously [7,8] or recently [21–23] been found to have critical protective roles against *F*. *tularensis* challenge. Interestingly, we identified four genes (IL-12rβ2, IL-27, IL-18bp, and CCR5) that follow the relative hierarchy of *in vitro* T cell activities in BALB/cByJ mice. Four more additional genes (IFN-γ, GM-CSF, T-bet, and SOCS-1) showed no differences among the splenocytes from the live vaccine groups, but they were consistently upregulated in comparison to those from the HK-LVS vaccine group. In addition, differences between mouse strains were revealed by the genes’ expression of TNF-α, IL-6, and IL-22. In contrast to C57BL/6J mice, these genes showed no differential expression between vaccine groups in BALB/cByJ mice. Among the genes analyzed, IL-12rβ2, IL-27, IL-18bp, IFN-γ, GM-CSF, SOCS-1 and T-bet, may represent the best candidates for a multivariate analysis that would discriminate T cell functions in response to different vaccines in both mouse strains. However, additional ongoing studies indicate slight different gene expression profiles when peripheral blood leukocytes derived from vaccinated C57BL/6J mice, instead of splenocytes, were used in co-cultures.

We next focused our attention on the analysis of B cell functions, to evaluate whether they affect immune responses in BALB/cByJ mice against the HK-LVS vaccine. Both wild type BALB/cByJ and BKO mice were fully protected against maximal lethal IP challenge when vaccinated with LVS, indicating that B cells are not necessary, and thus that T cells are likely sufficient, when the vaccine induces a strong T cell response. The lack of B cells made the BKO mice more susceptible to lethal LVS challenge when they were vaccinated with LVS-G, LVS-R, or HK-LVS (Table 1). The overall survival rates resembled that of C57BL/6J wild type mice, and a substantial drop in survival compared to BALB/cByJ wild type mice was observed in mice vaccinated with HK-LVS. Although the lack of B cells may have impacts also on the T cell functions, the T cell immune response against LVS-derived vaccines appeared to be overall similar between BALB/cByJ and BKO mice. The subtle differences between T cell functions of BALB/cByJ and BKO mice are likely to have minimal effects on the reduced protection against LVS challenge. The most interesting differences were represented by the lower gene expression of IFN-γ IL-12rβ2, and GM-CSF in the LVS-vaccinated BKO group compared to the LVS-G and LVS-R vaccinated groups. Whether the expression *in vitro* of these genes is due to some cooperative function between T and B cells is not yet clear. However, production of secreted IFN-γ seems not to be affected, nor is NO activity (Fig 2).

B cell functions, not related to antibody production, are important in primary and secondary protective immunity against *F*. *tularensis* LVS in C57BL/6J mice [17]. In addition, protective roles for antibodies has been demonstrated by transferring serum derived from LVS-vaccinated mice [11,28,40], from HK-LVS-vaccinated mice [41], from SchuS4-infected mice [42] or from LVS LPS-vaccinated mice [43] into mice at the time of or shortly before lethal challenge. Moreover, antibodies derived from LVS-vaccinated Fischer 344 rats protected against intratracheal challenge with a low dose of *F*. *tularensis* SchuS4 [18]. The identification and quantification of protective antibodies could lead to an informative multivariate analysis, by combining the antibody titers with T cell correlates, and thus account for both B and T cell immune responses. However, our findings to establish the protective role of antibodies when BALB/cByJ mice were vaccinated with sub-optimal vaccines are not consistent with a quantitative role for total anti-LVS antibodies. When measuring anti-LVS total IgG, we observed somewhat higher but variable antibodies titers in BALB/cByJ compared to C57BL/6J mice, either when vaccinated with HK-LVS and LVS-G, at 6 weeks following vaccination and after challenge (Fig 3, S3 Table, and S4 Table). Thus, whether these higher anti-*Francisella* antibody titers were sufficient to protect HK-LVS-vaccinated mice, when challenged with a lethal dose of LVS, remains unclear.

Similar contradictory results were obtained when we vaccinated BALB/cByJ mice with different doses of HK-LVS. Antibody titers were slightly higher in the mice vaccinated with higher dose, and minimal with lower vaccination doses, suggesting a role of the antibodies in survival and delayed time of death. However, we observed a high mouse-to-mouse variability that did not correlate with survival (Fig 5). Indeed, mice that survived the LVS challenge did not necessarily exhibit higher antibody production after primary vaccination. It is possible that the total anti-*Francisella* IgG may include one or a few protective antibodies, which, although important, may represent only a small fraction and therefore may have little impact on the total antibody titers. For instance, protective antibodies against *F*. *tularensis* outer membrane protein A [44] or against epitopes of LVS LPS [45] have been identified, and their presence has been demonstrated in human serum [46]. We therefore investigated the protein production and antigenic content of the LVS-derived vaccines. Whereas LVS, LVS-G, and LVS-R protein expression appeared to be comparable, the HK-LVS vaccine showed a reduction of most of the proteins (Fig 4), suggesting that the heat treatment of LVS degrades the proteins. However, many proteins are still recognized by the sera from LVS-vaccinated mice, suggesting that the HK-LVS vaccine still retains antigenic properties which induce humoral immune responses (Fig 4, panel B). Similarly, sera derived from HK-LVS-vaccinated mice reacted with proteins of all vaccines, although to a lesser extent than with those of HK-LVS and LVS-R (Fig 4, panel C). Of note, the methods used here detected small differences, but these studies were not exhaustive. Previous studies have demonstrated that LVS-vaccinated BALB/cByJ and C57BL/6J mice generated antibodies that recognized different panels of proteins, several of which were unique to BALB/cByJ and thus associated with successful vaccination of this mouse strain [47,48]. Taken together, our studies indicate that immune B cells’ function play a more important role in BALB/cByJ mice than in C57BL/6J mice. Whether this role is due to regulatory functions of B cells, such as antigen presentation, cytokine secretion, production of natural IgM, and/or production of anti-*F*. *tularensis* specific antibodies, is still unclear. Understanding of these characteristics is essential to fully evaluate novel vaccines, derive potential correlates of protection, and ultimately to identify animal models that best can bridge to human subjects. More importantly, our findings support the validity of this approach to identify and monitor potential T cell immune correlates of protection.

## Supporting Information

S1 Fig TNF-α and IL-12 production exhibit patterns that mostly correlate to those of *in vitro* LVS replication. Supernatants from co-cultures described in Fig 1 using splenocytes of BALB/cByJ mice were collected after two days of co-culture, and separated from cells for analyses of TNF-α, (Panel A), IL-12 p40 (Panel B) and IL-6 (Panel C) by ELISA. Concentrations were calculated using standard curves as reference. Values shown are the mean concentration in pg/ml (TNF-α) or ng/ml (IL-12 p40 and IL-6) ± standard deviation of triplicate samples. Results shown are from one representative experiment of seven independent experiments of similar design and outcome. Brackets indicate a significant difference (*P* < 0.05) between amounts of TNF-α and IL-12 produced in co-cultures. There were no significant differences in TNF-α and IL-12 production between the co-cultures using LVS-immune cells and the co-cultures using LVS-G-immune cells, nor between the co-cultures using LVS-G-immune cells and the co-cultures using LVS-R-immune cells (Panels A and B). There were no significant differences in IL-6 production between the co-cultures using LVS-immune cells and the co-cultures using LVS-G-, LVS-R- or HK-LVS- immune cells (Panel C).
(TIF)

S1 Table Distribution of cell subpopulations used for *in vitro* co-culture studies. Single cell preparations from the indicated vaccinated mice were stained for surface markers and analyzed by flow cytometry. Cells were initially gated for CD45 and live cells, and then further characterized with the indicated markers. Panel A shows data from analyses of cells isolated from the indicated vaccinated mice and used for co-culture experiments. Panel B shows data from the same cells recovered after 48 hours co-culture. Values represent average of percents from seven independent experiments.
(DOC)

S2 Table Relative gene expression of T- and B-cell activation factors in co-cultures using splenocytes from differentially vaccinated C57BL/6J and BALB/cByJ mice. Real-time PCR was performed using the T- and B-cell activation profiler array. Values indicate median fold change of the indicated genes, compared to naive cells; the values were derived from analyses of splenocytes of C57BL/6J and BALB/cByJ mice, and were calculated using data from four independent experiments. The list includes genes that were either differentially expressed among vaccine groups or between mouse strains. T- and B-cell related factors indicate genes involved mostly in the activation, proliferation, and differentiation of T- and B-cell, respectively. Others indicate factors involved mostly in non-T or non-B cell activities.
(DOC)

S3 Table Anti-LVS total IgG titers of vaccinated mice. Pooled sera from five mice for each vaccine group were obtained at the indicated time points and analyzed for anti-LVS total IgG. Those experiments were repeated four times in both BALB/cByJ and C57BL/6J mice. Shown are medians and ranges of antibody titers obtained using data from the four replicate experiments.
(DOC)

S4 Table Anti-LVS IgM and IgG isotypes titers of vaccinated mice. Pooled sera from five mice for each vaccine group were obtained at the indicated time points and analyzed for anti-LVS total IgM and anti-LVS IgG isotypes. Shown are antibody titers obtained from two replicate experiments. *Titers for sera obtained from BALB/cByJ mice. ^#^Titers for sera obtained from C57BL/6J mice.
(DOC)
