# Supplementary figures and images for: Francisella tularensis Vaccines Elicit Concurrent Protective T- and B-Cell Immune Responses in BALB/cByJ Mice
Source: PLoS One. 2015 May 14;10(5):e0126570. doi: 10.1371/journal.pone.0126570 (PMC4431730; doi:10.1371/journal.pone.0126570)

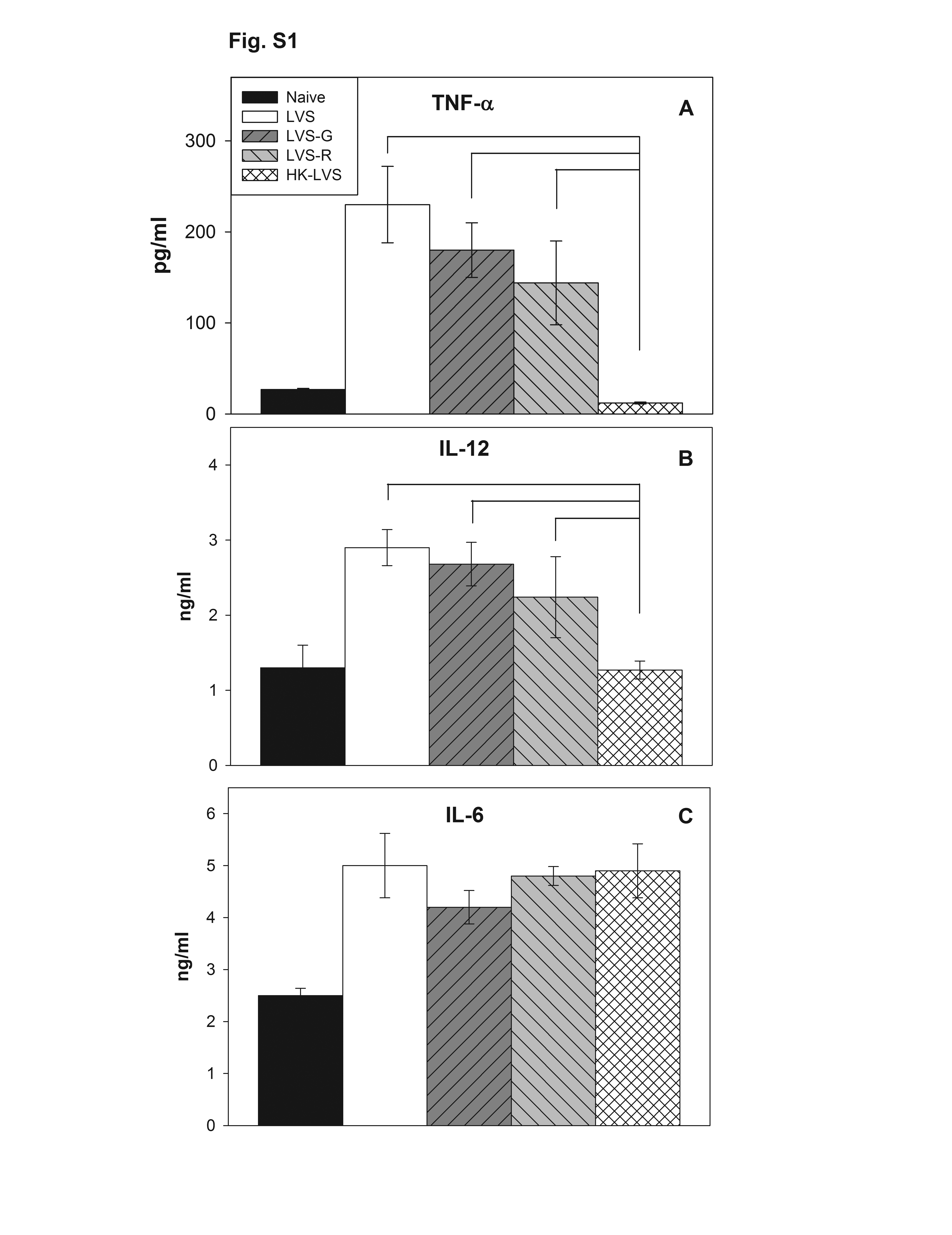

Supplement: S1 Fig — Supernatants from co-cultures described in Fig 1 using splenocytes of BALB/cByJ mice were collected after two days of co-culture, and separated from cells for analyses of TNF-α, (Panel A), IL-12 p40 (Panel B) and IL-6 (Panel C) by ELISA. Concentrations were calculated using standard curves as reference. Values shown are the mean concentration in pg/ml (TNF-α) or ng/ml (IL-12 p40 and IL-6) ± standard deviation of triplicate samples. Results shown are from one representative experiment of seven independent experiments of similar design and outcome. Brackets indicate a significant difference (P < 0.05) between amounts of TNF-α and IL-12 produced in co-cultures. There were no significant differences in TNF-α and IL-12 production between the co-cultures using LVS-immune cells and the co-cultures using LVS-G-immune cells, nor between the co-cultures using LVS-G-immune cells and the co-cultures using LVS-R-immune cells (Panels A and B). There were no significant differences in IL-6 production between the co-cultures using LVS-immune cells and the co-cultures using LVS-G-, LVS-R- or HK-LVS- immune cells (Panel C). (TIF) [file pone.0126570.s001.TIF]
