# Supplementary material for: Francisella tularensis Vaccines Elicit Concurrent Protective T- and B-Cell Immune Responses in BALB/cByJ Mice
Source: PLoS One. 2015 May 14;10(5):e0126570. doi: 10.1371/journal.pone.0126570 (PMC4431730; doi:10.1371/journal.pone.0126570)
Supplement: S1 Table — Single cell preparations from the indicated vaccinated mice were stained for surface markers and analyzed by flow cytometry. Cells were initially gated for CD45 and live cells, and then further characterized with the indicated markers. Panel A shows data from analyses of cells isolated from the indicated vaccinated mice and used for co-culture experiments. Panel B shows data from the same cells recovered after 48 hours co-culture. Values represent average of percents from seven independent experiments. (DOC) [file pone.0126570.s002.doc]

**S1 Table**

**A**

|  | **Markers** | **LVS** | **LVS-G** | **LVS-R** | **HK-LVS** | **Naive** |
| --- | --- | --- | --- | --- | --- | --- |
| Live leucocytes | CD45^+^ Live/dead | 89.2 | 88.5 | 87.4 | 85.4 | 88.0 |
| B cells | CD19^+^ B220^+^ | 35.3 | 35.3 | 33.4 | 34.4 | 38.0 |
| T cells CD4 | TCRβ^+^ CD4^+^ | 35.6 | 36.1 | 36.9 | 36.1 | 34.0 |
| T cells CD8 | TCRβ^+^ CD8^+^ | 16.0 | 15.7 | 14.9 | 15.1 | 14.1 |
| NK | NK1.1^+^ TCRβ^-^ CD19^-^ | 1.4 | 1.6 | 1.6 | 1.5 | 2.1 |
| DC | CD11c^+^ TCRβ^-^ CD19^-^ | 0.8 | 0.8 | 1.2 | 0.9 | 1.0 |
| NK T | TCRβ^+^ NK1.1^+^ CD19^-^ | 0.9 | 0.9 | 1.0 | 0.9 | 0.9 |
| PMN | CD11b^+^ Gr1^+^ TCRβ^-^ CD19^-^ | 1.0 | 1.0 | 1.0 | 1.1 | 1.1 |
| Macs | CD11b^+^ Gr1^-^ TCRβ^-^ CD19^-^ | 1.9 | 2.2 | 3.2 | 3.1 | 3.0 |

**B**

|  | **Markers** | **LVS** | **LVS-G** | **LVS-R** | **HK-LVS** | **Naive** |
| --- | --- | --- | --- | --- | --- | --- |
| Live leucocytes | CD45^+^ Live/dead | 68.1 | 69.2 | 65.2 | 56.7 | 64.5 |
| B cells | CD19^+^ B220^+^ | 21.5 | 29.3 | 40.9 | 43.3 | 43.2 |
| T cells CD4 | TCRβ^+^ CD4^+^ | 51.1 | 46.9 | 38.6 | 39.8 | 40.0 |
| T cells CD8 | TCRβ^+^ CD8^+^ | 18.9 | 16.5 | 12.2 | 10.0 | 10.3 |
| NK | NK1.1^+^ TCRβ^-^ CD19^-^ | 0.7 | 0.6 | 0.5 | 0.4 | 0.7 |
| DC | CD11c^+^ TCRβ^-^ CD19^-^ | 0.5 | 0.7 | 1.0 | 0.3 | 0.3 |
| NK T | TCRβ^+^ NK1.1^+^ CD19^-^ | 0.4 | 0.2 | 0.4 | 0.3 | 0.4 |
| PMN | CD11b^+^ Gr1^+^ TCRβ^-^ CD19^-^ | 0.1 | 0.1 | 0.1 | 0.1 | 0.1 |
| Macs | CD11b^+^ Gr1^-^ TCRβ^-^ CD19^-^ | 0.4 | 0.6 | 0.4 | 0.4 | 0.5 |
