# Supplementary material for: Francisella tularensis Vaccines Elicit Concurrent Protective T- and B-Cell Immune Responses in BALB/cByJ Mice
Source: PLoS One. 2015 May 14;10(5):e0126570. doi: 10.1371/journal.pone.0126570 (PMC4431730; doi:10.1371/journal.pone.0126570)
Supplement: S2 Table — Real-time PCR was performed using the T- and B-cell activation profiler array. Values indicate median fold change of the indicated genes, compared to naive cells; the values were derived from analyses of splenocytes of C57BL/6J and BALB/cByJ mice, and were calculated using data from four independent experiments. The list includes genes that were either differentially expressed among vaccine groups or between mouse strains. T- and B-cell related factors indicate genes involved mostly in the activation, proliferation, and differentiation of T- and B-cell, respectively. Others indicate factors involved mostly in non-T or non-B cell activities. (DOC) [file pone.0126570.s003.doc]

**S2 Table**

|  |  | **C57BL/6J** | | | **BALB/cByJ** | | |
| --- | --- | --- | --- | --- | --- | --- | --- |
|  |  | **LVS** | **LVS-R** | **HK-LVS** | **LVS** | **LVS-R** | **HK-LVS** |
|  | **CD1d1** | 1.2 | 1.3 | 1.2 | 2.4 | 1.7 | 1.0 |
|  | **Irf-4** | 1.0 | 0.8 | 1.2 | 2.2 | 1.7 | 1.5 |
|  | **Prlr** | 0.6 | 1.2 | 1.9 | 2.1 | 2.3 | 0.7 |
| **T cell** | **Pdcd1lg2** | 1.9 | 1.5 | 1.3 | 4.5 | 2.8 | 1.1 |
| **related** | **IL-15** | 0.9 | 1.2 | 1.0 | 4.0 | 1.5 | 0.9 |
| **factors** | **IL-27** | 4.1 | 2.6 | 1.0 | 9.3 | 3.6 | 1.5 |
|  | **Gadd45g** | 1.9 | 1.6 | 1.0 | 2.4 | 1.9 | 1.1 |
|  | **CD28** | 0.9 | 0.9 | 0.8 | 2.0 | 1.6 | 0.7 |
|  | **IFN-γ** | 36 | 41 | 1.9 | 26 | 9.9 | 1.5 |
|  | **Igbp1b** | 0.4 | 0.9 | 12 | 4.5 | 3.7 | 0.7 |
| **B cells** | **Cdkn1a** | 2.1 | 1.2 | 1.0 | 7.3 | 2.7 | 1.8 |
| **related** | **Nkx2-3** | 0.8 | 0.7 | 1.6 | 1.8 | 2.3 | 0.6 |
| **factors** | **Inha** | 0.7 | 1.2 | 3.0 | 1.4 | 1.6 | 1.0 |
|  | **Rag1** | 0.6 | 1.3 | 3.1 | 1.7 | 2.2 | 0.9 |
|  | **IL-11** | 1.1 | 1.3 | 5.3 | 2.7 | 2.7 | 2.0 |
|  | **GM-CSF2** | 1.5 | 2.0 | 0.8 | 2.4 | 4.1 | 1.0 |
|  | **Rgs1** | 1.7 | 1.1 | 0.9 | 3.4 | 2.6 | 0.9 |
| **Others** | **Tlr4** | 1.2 | 1.2 | 1.5 | 6.1 | 2.4 | 1.0 |
|  | **CD93** | 0.6 | 0.5 | 1.3 | 2.4 | 3.1 | 1.2 |
|  | **Egr1** | 1.4 | 1.0 | 1.5 | 3.3 | 1.8 | 1.2 |
