# Supplementary material for: Francisella tularensis Vaccines Elicit Concurrent Protective T- and B-Cell Immune Responses in BALB/cByJ Mice
Source: PLoS One. 2015 May 14;10(5):e0126570. doi: 10.1371/journal.pone.0126570 (PMC4431730; doi:10.1371/journal.pone.0126570)
Supplement: S3 Table — Pooled sera from five mice for each vaccine group were obtained at the indicated time points and analyzed for anti-LVS total IgG. Those experiments were repeated four times in both BALB/cByJ and C57BL/6J mice. Shown are medians and ranges of antibody titers obtained using data from the four replicate experiments. (DOC) [file pone.0126570.s004.doc]

**S3 Table**

|  | **Two weeks after vaccination** | | **Six weeks after vaccination** | | | **Three days after challenge** | |
| --- | --- | --- | --- | --- | --- | --- | --- |
| **Group** | **BALB/c** | **C57BL/6J** | **BALB/c** | | **C57BL/6J** | **BALB/c** | **C57BL/6J** |
| LVS | 800 | 3840 | 3840 | 7680 | | 1920 | 3840 |
|  | 320-1280 | 640-10240 | 1280-10240 | 1280-10240 | | 1280-10240 | 1280-10240 |
| LVS-G | 640 | 640 | 1280 | 640 | | 1280 | 640 |
|  | 160-1280 | 40-2560 | 640-5120 | 320-2560 | | 640-2560 | 160-640 |
| LVS-R | 90 | 340 | 200 | 240 | | 80 | 50 |
|  | <20-160 | <20-1280 | <20-1280 | 80-2560 | | 20-320 | <20-320 |
| HK-LVS | 40 | 30 | 400 | | 50 | 200 | 25 |
|  | <20-640 | <20-40 | 160-10240 | | <20-160 | 40-640 | <20-160 |
