# Supplementary material for: Francisella tularensis Vaccines Elicit Concurrent Protective T- and B-Cell Immune Responses in BALB/cByJ Mice
Source: PLoS One. 2015 May 14;10(5):e0126570. doi: 10.1371/journal.pone.0126570 (PMC4431730; doi:10.1371/journal.pone.0126570)
Supplement: S4 Table — Pooled sera from five mice for each vaccine group were obtained at the indicated time points and analyzed for anti-LVS total IgM and anti-LVS IgG isotypes. Shown are antibody titers obtained from two replicate experiments. *Titers for sera obtained from BALB/cByJ mice. #Titers for sera obtained from C57BL/6J mice. (DOC) [file pone.0126570.s005.doc]

**S4 Table**

|  |  | **Two weeks after vaccination** | | **Six weeks after vaccination** | | **Three days after challenge** | |
| --- | --- | --- | --- | --- | --- | --- | --- |
|  | **Group** | **BALB/c** | **C57BL/6J** | **BALB/c** | **C57BL/6J** | **BALB/c** | **C57BL/6J** |
|  | LVS | 1280, 2560 | 80, 2560 | 80, 2560 | 40, 80 | 80, 640 | 20, 40 |
| IgM | LVS-G | 640, 1280 | 80, 640 | 80, 640 | 80, 320 | <20, 40 | 80, 160 |
|  | LVS-R | <20, 40 | 20, 40 | 20, 40 | <20, 80 | 20, 40 | 20, 40 |
|  | HK-LVS | 640, 1280 | 160, 640 | 160, 640 | 40, 80 | 80, 640 | 40, 80 |
|  | LVS | <20, 10240 | <20, 80 | 640, 5120 | 1280, 5120 | 5120, 5120 | 40, 1280 |
| IgG_1_ | LVS-G | 320, 10240 | <20, 40 | 640, 10240 | <20, 320 | 640, 20480 | <20, 80 |
|  | LVS-R | 320, 1280 | <20, 160 | 80, 10240 | 20, 60 | <20, 160 | <20, 80 |
|  | HK-LVS | 20, 2560 | <20, <20 | 1280, 5120 | <20, 40 | 320, 2560 | <20, 160 |
|  | LVS | 40, 160 | 5120, 10240 | 160, 640 | 20480, 2480 | 320, 1280 | 2560, 10240 |
| IgG_2b_ | LVS-G | 20, 320 | 1280, 5120 | 160, 160 | 640, 1280 | 80, 160 | 320, 640 |
|  | LVS-R | <20, 160 | 1280, 2560 | 20, 80 | 320, 640 | <20, 20 | 80, 160 |
|  | HK-LVS | <20, 320 | <20, 40 | 20, 320 | 160, 320 | 20, 320 | 160, 320 |
|  | LVS | 40, 40 | 20, 40 | 320, 320 | 320, 640 | 160, 320 | 40, 80 |
| IgG_3_ | LVS-G | 40, 80 | 80, 80 | 160, 640 | 640, 640 | 40, 320 | 640, 640 |
|  | LVS-R | <20, 40 | <20, 160 | <20, 40 | 20, 20 | <20, 40 | <20, 20 |
|  | HK-LVS | 40, 40 | 40, 80 | 80, 640 | 160, 320 | 80, 320 | 160, 160 |
| IgG_2a_* | LVS | 320, 1280* | 640, 640^#^ | 5120, 5120* | 5120, 5120^#^ | 5120, 5120* | 640, 20480^#^ |
| or | LVS-G | 160, 320* | 80, 1280^#^ | 640, 640* | 80, 320^#^ | 320, 320* | <20, 160^#^ |
| IgG_2c_^#^ | LVS-R | <20, 80* | 320, 640^#^ | 80, 320* | 40, 160^#^ | 40, 160* | 20, 40^#^ |
|  | HK-LVS | <20, 1280* | <20, <20^#^ | <20, 160* | <20, 20^#^ | <20, 160* | <20, <20^#^ |
